# The Importance of Humidity in the Relationship between Heat and Population Mental Health: Evidence from Australia

**DOI:** 10.1371/journal.pone.0164190

**Published:** 2016-10-11

**Authors:** Ning Ding, Helen L. Berry, Charmian M. Bennett

**Affiliations:** 1 Research Center for Medical Education, China Medical University, Shenyang, Liaoning Province, China; 2 ANU Climate Change Institute, The Australian National University, Acton, Australia; 3 National Centre for Epidemiology and Population Health, Research School of Population Health, The Australian National University, Acton, Australia; University of Queensland, AUSTRALIA

## Abstract

Despite many studies on the effects of heat on mental health, few studies have examined humidity. In order to investigate the relationship among heat, humidity and mental health, we matched data from the Social, Economic and Environmental Factors (SEEF) project with gridded daily temperature and water vapour pressure data from the Australian Bureau of Meteorology. Logit models were employed to describe the associations among heat (assessed using temperature, °C), humidity (assessed using vapour pressure, hPa) and two measures of mental health, (i) high or very high distress (assessed using K10 scores ≥ 22) and (ii) having been treated for depression or anxiety. We found a one-unit increase in temperature and vapour pressure was associated with an increase in the occurrence of high or very high distress by 0.2% (*p* < 0.001, 99% CI: 0.1–0.3%) and 0.1% (*p* < 0.001, 99% CI: 0.0–0.3%) respectively. However, when humidity rose to the 99^*th*^ percentile of the sample, the estimated marginal effect of heat was more than doubled (0.5%, *p* < 0.001, 99% CI: 0.2–0.7%). Neither heat nor humidity was related to having been treated for depression or anxiety in the last month. Humidity compounds the negative association between hot weather and mental health and thus should be taken into account when reforming the health care system to respond to the challenge of climate change.

## Introduction

Evidence has accumulated that increasingly variable and severe weather events (such as heat waves and droughts) over the last several decades have caused substantial harm to human wellbeing [1] and directly and indirectly threatened population mental health [2–5]. Variability in temperature, especially more intense, frequent and longer-lasting heat waves, have not only caused substantial excess deaths [6] but also deteriorations in population mental health [7–9]. This is a significant concern in the context of global warming (especially in developing countries where mental health services are woefully inadequate and hot weather is commonplace) because psychiatric disorders are the leading cause of global burden of disease [10]. Further, since 1973, the world’s atmosphere has been getting moister by roughly 0.1 grams of water vapour per kilogram of air per decade [11]. This has not attracted much attention from researchers of mental health despite a well-documented compound association between humidity and physical health [12]. It is well-understood that perspiring is the body’s primary physiological response to maintain core temperature when under heat stress, and that this process is inhibited in very humid conditions as the air approaches saturation point. Saturated air does not evaporate moisture from the skin surface very effectively, which reduces the efficiency of sweating to reduce the heat load. Thus, we could expect extreme heat to have worse effects on mental health when humidity is high. However, to our knowledge, there have been no studies of the possible interactive effects on mental health of heat together with humidity. This makes investigation of the combined effects of heat and humidity on mental health an urgent research priority.

We sought to examine the association between mental health, heat and humidity systematically by undertaking a case study in New South Wales, an Australian state of 7.5 million people in which there is sufficient climate zone variability and extensive and reliable heat, humidity and population mental health screening data. We aimed to make two key contributions: first, to characterise and provide a reliable estimation of the associations between heat, humidity and population mental health; second, to use two approaches to measuring mental health status in the last month (presence of symptoms of anxiety and depression; and whether a person had been treated for either condition). This permitted consideration of service need (approximated by presence of symptoms) relative to service access (approximated by service received) to elucidate possible climate change-related reforms to health care systems that might be necessary for future heat-related mental health demand.

## Methods

### Data and measures

Data were drawn from two sources: the Social, Economic and Environmental Factors (SEEF) sub-study of the Sax Institute’s 45 and Up Study [13] (www.saxinstitute.org.au) and the Australian Government Bureau of Meteorology gridded daily temperature and water vapour pressure data [14].

The 45 and Up Study is a large-scale cohort study of 267,151 residents aged 45 and over of the south-east Australian state of New South Wales, Australia’s most populous state, representing approximately 10% of the State population in this age group. The conduct of the 45 and Up Study was approved by the University of New South Wales Human Research Ethics Committee (Ref no.: HREC 05035). Between 2006 and 2008, 100,000 randomly selected respondents already enrolled in the 45 and Up Study were invited to participate in an additional sub-study called the Social, Economic and Environmental Factors (SEEF) study, granted ethical approval by the University of Sydney Human Research Ethics Committee (Ref no.: 10-2009/12187). A total of 60,404 respondents completed the SEEF questionnaire which covered a range of socio-demographic, lifestyle, income, employment and social circumstances as well as physical and mental health (details below). The SEEF sub-study also collected data on gender, age, educational attainment, relationship status, language other than English used at home, personal income, retirement status and employment status. Importantly, the date on which the participant completed the SEEF questionnaire was collected, so that their data could be matched with weather data up to and including that date (details below). The majority of participants completed their questionnaires in springs (between October and December) and the distribution of participants over months of the year is shown in S1 Table.

The SEEF sub-study contained the Kessler ten-item measure (K10) measuring symptoms of nonspecific psychological distress in the last four weeks [15]. Each item was scored on a five-point scale from 1 = ‘none of the time’ to 5 = ‘all of the time’. Final summed scores had a possible range of 10-50 with higher scores indicating higher levels of distress. Participants were also asked whether they had been treated for depression or anxiety over the same period (four weeks). The 45 and Up Study had previously collected information about participants’ pre-study diagnoses of physical illnesses (e.g., heart failure, skin cancer) and mental illnesses (depression, anxiety) which we included in our dataset. We therefore had five measures of mental health: general psychological distress over the last four weeks (K10 score, hereafter ‘distress’); presence of high or very high levels of psychological distress (high levels: 22 ≤ K10 ≤ 29; very high levels: K10 ≥ 30) [16, 17]; being currently treated for depression or anxiety (neither = 0, either or both = 1); history of depression (yes = 1, no = 0); history of anxiety (yes = 1, no = 0). The mean K10 score by month of the year when participants completed questionnaires is shown in S1 Fig, suggesting no evidence of monthly differences in the response variable.

We used the Australian Government Bureau of Meteorology gridded daily temperature and vapour pressure data at a resolution of 0.05° × 0.05° (about 5km × 5km). The temperature and vapour pressure in each grid were not measured directly, but were produced based on *in situ* observations with the help of robust topography-resolving analysis methods with necessary and desirable adjustments when applying in Australia. The accuracy of this approach to spatial analysis has been demonstrated by using verification against weather station observations [18]. We matched each Statistical Local Area (SLA), an Australian Standard Geographical Classification (ASGC) defined area, in New South Wales with the meteorological grid which covered its centroid. The SLA is the smallest spatial unit for which most government-collected data are available. There are 199 SLAs in NSW. The area of each SLA varies from 4.2 *km*^2^ to nearly 100,000 *km*^2^ and the populations of SLAs can vary from hundreds to more than 100,000 persons, with an average of 35,232 in 2008 [19]. We obtained the maximum daily temperature in Celsius degrees (°C) and the vapour pressure at 3 p.m. in hPas (the approximate vapour pressure when temperature reached its daily maximum) for each SLA for every day of the period between 2006 and 2008 when the SEEF data were collected (no missing data).

Vapour pressure is defined as the pressure exerted by a vapour in thermodynamic equilibrium with its condensed phases (solid or liquid) at a given temperature, so the more humid, the more water molecules in air and the higher the vapour pressure. In the present study, we used vapour pressure, one absolute measure, to approximate humidity, because relative measures of humidity are a function of both water vapour content and air temperature so that the use of such measures would severely bias and even change the sign of the estimate of health effect of heat. In this study, to measure heat and humidity, we computed the average daily maximum temperature and the water vapour pressure at 3p.m. respectively for the last four weeks for each SLA. The four-week period was selected to correspond with the time period of the ‘previous four weeks’ over which distress was measured.

Date of completion and SLAs were only available for 53,464 of the 60,404 SEEF respondents who completed the questionnaire. We selected these respondents and linked their SEEF data with the heat and humidity data for their SLA for the four weeks immediately preceding the date on which they completed the survey. The respondents in the final sample came from all 199 SLAs. A small proportion (0.6%) of mental health data were missing, leaving a final sample of 53,144 participants for analysis.

### Statistical analysis

In the dataset of 53,144 SEEF participants, there were very few missing values on any of the other variables (no more than 1.5%, except for personal income which had about 8.5% missing values). To be able to utilise the full sample, these missing values were estimated using multiple imputation. Although studies suggest only three imputations are needed [20], from which mean values are derived, we conducted ten imputations to ensure the accuracy of the final imputed values. Sensitivity analyses comparing estimates derived using the datasets with and without imputation indicated no significant differences between the two; all analyses were therefore conducted using the larger dataset with imputed values.

After controlling for confounders (see below), we conducted four Logit regression models to estimate the correlations between mental health and weather conditions. In model 1, we used one principle predictor – heat, which was measured by the average daily maximum temperature in last four weeks, to predict mental health. In model 2, we used only humidity, which was measured by the average water vapour pressure at 3 p.m. in last four weeks, to predict mental health. In model 3, we used heat and humidity together and, in model 4, we predicted mental health using heat, humidity and an interaction term for heat and humidity. In addition to the estimated regression coefficients, to make the results more substantively meaningful to readers, we have also reported the marginal effects at the means [21].

We controlled in all analyses for a wide range of variables that could have confounded the relationship between heat, humidity and mental health: the participant’s history of physical and mental health, demographic characteristics and socio-economic factors. Physical health history was measured by the number of physical diagnoses the participant had ever had; mental health history controlled for whether the respondent had been diagnosed with depression and/or anxiety in the 12 months before the survey. Demographic and socio-economic factors were gender (reference category = female), age, age squared and the interaction of each of these with gender, educational attainment (dummy variables for ‘did not complete secondary education’; ‘Year 12, trade or apprenticeship’; ‘diploma or certificate’; reference category = ‘bachelor’s degree or above’), relationship status (dummy variables for widowed, married, separated/divorced, and living together as a couple, reference category = single), language other than English used at home (reference category = English), personal income (dummy variables for AU$10,000 – 19,999, AU$20,000 – 39,999, AU$40,000 – 69,999 and AU$70,000 or more, reference category = less than AU$10,000), retirement status (reference category = retired), employment status (reference category = unemployed) and population dispersion in each Statistical Local Area (proportion residing in remote areas and proportion residing in regional areas; remainder are urban). We introduced age and its quadratic term simultaneously to capture the possible nonlinear association between age and distress.

In addition, to test for a non-linear relationship in the association between mental health and weather conditions, we computed a second version of models 1 and 2 above (models 1′ and 2′) in which we created four categorical variables each for temperature and vapour pressure based on their values at the 90^*th*^, 95^*th*^, 97^*th*^ and 99^*th*^ percentiles. For temperature, these corresponded to 25.0°C, 26.3°C, 26.8°C and 27.8°C and 18.1 hPa, 19.2 hPa, 19.6 hPa and 20.4 hPa vapour pressure.

Finally, we implemented a series of robustness checks: we 1) re-estimated all the models using heat and humidity based on the daily average temperature and average vapour pressure respectively (vs the approximate maximums); 2) introduced into the regression equations a variable indicating whether a SLA was located in a coastal or inland region (to inspect the dataset for coastal SLAs in NSW, see the report of Australian Institute of Health and Welfare [22]); and 3) introduced the average solar exposure in last four weeks in *MJ*/*m*^2^s into the regression equations. The results confirmed the robustness of our estimations (See S2, S3 and S4 Tables).

All analyses and multiple imputations were carried out using STATA 13 [23].

## Results

Descriptive statistics are presented in Table 1 which shows the proportions of respondents by mental health and socio-economic categories, split by whether they completed the questionnaire during a month that recorded a level of vapour pressure that was higher or lower than the sample median (14.4 hPa). The sample median was slightly higher than the annual median (13.2 hPa) since most of the surveys were completed during spring, which is typically more humid than the annual average. High-humidity conditions were associated with lower levels of educational attainment, lower personal income and greater probability of being in retirement. More than 6% of participants reported high or very high distress (K10 ≥ 22) in the last month, while about 8% reported having been treated for depression or anxiety over the same period. Those in high-humidity conditions were very slightly and significantly more likely than their peers to report high or very high distress but there was no difference between the groups for whether they had been treated for anxiety or depression.

**Table 1:** Proportions of respondents by socio-demographic characteristics and humidity status.

	Percent			*P*-value
	All (N = 53,144)	High humidity^*a*^ (n = 26,574)	Low humidity^*b*^ (n = 26,570)	
High or very high distress (K10 ≥ 22)	6.4	6.7	6.2	0.04
Treated for depression or anxiety in last month (yes)	8.0	7.9	8.1	0.31
Diagnosed with depression one year previous (yes)	11.8	11.8	11.8	0.95
Diagnosed with anxiety one year previous (yes)	7.9	8.1	7.7	0.11
Male	46.7	46.4	47.0	0.16
Highest education attainment				**<0.001**
Did not complete secondary education	9.4	9.7	9.1	
Year 12, trade or apprenticeship	42.4	42.9	41.9	
Diploma or Certificate	22.58	22.67	22.5	
Bachelor’s degree or above	25.62	24.76	26.5	
Relationship status				0.16
Single	5.86	5.71	6.0	
Widowed	9.9	9.7	10.0	
Married	71.4	71.4	71.4	
Divorced or separated	8.0	8.1	7.9	
De facto	4.9	5.1	4.7	
Personal income				**<0.001**
Less than AU$10,000	8.9	8.9	8.9	
AU$10,000 – 19,999	23.0	24.0	22.0	
AU$20,000 – 39,999	26.3	26.8	25.8	
AU$40,000 – 69,999	17.9	17.1	18.7	
AU$70,000 or more	16.7	15.5	17.8	
Refused to answer	7.3	7.8	6.7	
Language other than English at home	7.0	7.0	7.0	0.95
Retired	46.6	48.6	44.5	**<0.001**
Unemployed	1.6	1.6	1.7	0.41

Notes:

^a^ High humidity means that average daily vapour pressure at 3 p.m. in the last four weeks was higher than the sample median of this measure (≥14.4 hPa);

^b^ Low humidity means that average daily vapour pressure at 3 p.m. in the last four weeks was lower than the sample median of this measure (<14.4 hPa).

The regression models predicting high or very high distress (Table 2) showed significant associations between heat, humidity and mental health such that, overall, the higher the heat and/or humidity, the greater the likelihood of high or very high distress (Models 1 and 2). Specifically, a one-unit increase in temperature or vapour pressure was associated with 0.2% (*p* < 0.001, 99% CI: 0.1-0.3%) and 0.1% (*p* < 0.001, 99% CI: 0.0-0.3%) increase in the likelihood of high or very high distress respectively. When introducing heat and humidity simultaneously (Model 3), the association between humidity and mental health became non-significant but higher temperatures remained significantly associated with poorer mental health. After introducing the interaction between heat and humidity (Model 4), the estimates for both heat and humidity remained significant but became negative; while the estimate for the interaction term was significant and positive. This suggests that the effect of humidity on distress is primarily that it amplifies the effect of heat: based on the marginal effects for the interaction model, a one-degree increase in temperature is associated with a 0.2% (*p* < 0.01, 99% CI: 0.0-0.4%) increase in the likelihood of high or very high distress while a similar increase in vapour pressure has no effect. Unlike the model for high or very high distress, the model predicting whether respondents had been treated for depression or anxiety shows that the estimated effects of both heat and humidity were non-significant. This indicates that, while hotter and more humid weather is associated with an increase in high or very high distress over short periods (in this case, the previous four weeks), it is not associated with an increase in rates of treatment over the same period.

**Table 2:** The associations between temperature (heat), vapour pressure (humidity) and mental health, Logit model for 53,144 adults aged over 45 from NSW, Australia.

	High or very high distress (K10 ≥ 22)		Whether treated for depression or anxiety in last month	
	Coef. (99% CI)	M. E. (99% CI)	Coef. (99% CI)	M. E. (99% CI)
Model 1^*a*^^*b*^				
Temperature	0.030 (0.010 – 0.051)**	0.2% (0.1% – 0.3%)**	0.004 (-0.017 – 0.025)	0.0% (-0.1% – 0.1%)
Model 2^*a*^^*b*^				
Vapour pressure	0.027 (0.006 – 0.049)**	0.1% (0.0% – 0.3%)**	-0.002 (-0.024 – 0.019)	0.0% (-0.1% – 0.1%)
Model 3^*a*^^*b*^				
Temperature	0.027 (-0.009 – 0.063)*	0.1% (-0.0% – 0.3%)*	0.019 (-0.018 – 0.056)	0.1% (-0.1% – 0.3%)
Vapour pressure	0.004 (-0.034 – 0.042)	0.0% (-0.2% – 0.2%)	-0.019 (-0.059 – 0.020)	-0.1% (-0.3% – 0.1%)
Model 4^*a*^^*b*^				
Temperature	-0.083(-0.169 – 0.004)	0.2% (0.0% – 0.4%)*	-0.045 (-0.135 – 0.044)	0.2% (-0.1% – 0.4%)
Vapour pressure	-0.201(-0.355 – -0.047)*	-0.1% (-0.3% – 0.1%)	-0.141 (-0.301 – 0.019)	-0.2% (-0.4% – 0.1%)
Temperature × Vapour pressure	0.010(0.002 – 0.015)**		0.005 (-0.001 – 0.012)	

Notes:

^a^ All models control for illness history (physical and mental), age, age squared and the interactions between age and gender and the interaction between age squared and gender, urbanicity/remoteness, labour force participation status, highest level of educational attainment, relationship status and use of language other than English at home (as a proxy for cultural background).

^b^ Because of the large sample size, significance values were set at:

* *p* <.01,

** *p* <.001.

**Fig 1:**
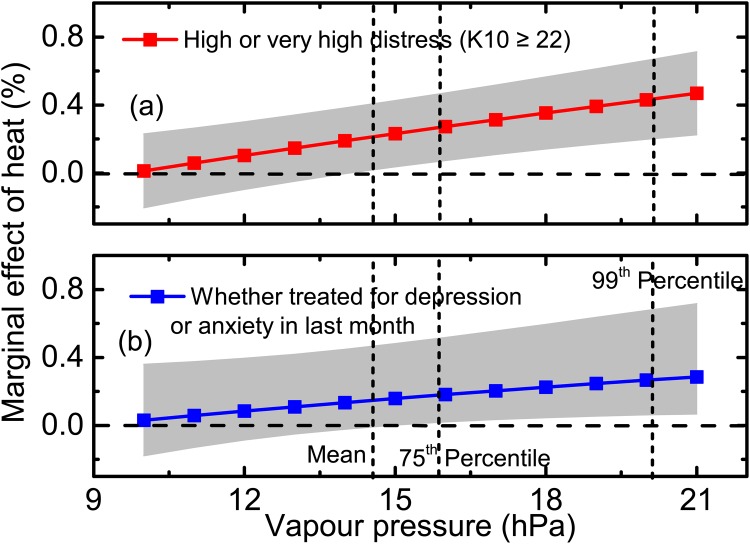
Marginal effects of heat on high or very high distress and on receiving treatment for depression or anxiety by humidity (vapour pressure) and their 99% confidential intervals, Logit model (Model 4), 53,144 adults aged over 45 from NSW, Australia.

Using the results from Model 4, we plotted the marginal effects of heat for different levels of humidity. Fig 1 shows the marginal effect of heat on mental health as humidity rises. As Fig 1(a) shows, the marginal effect of heat on high or very high distress rose monotonically with increasing humidity, producing a significant worsening of distress even when humidity was the approximate mean of its distribution in our dataset (i.e., when vapour pressure was about 14 hPa) reflecting the results reported in Table 2. But when humidity was high – at the 99^*th*^ percentile (where vapour pressure was about 21 hPa) – the marginal effect of heat rose to 0.5% (*p* < 0.001, 99% CI: 0.2-0.7%). This more than doubled the size of the effect of heat when humidity was at the sample mean.

Although the trend for the effect of heat and humidity on whether respondents had been treated for depression or anxiety in the last month was the same as for high or very high distress, the marginal effect of heat for given levels of humidity was much smaller, and non-significant, until vapour pressure rose above 16 hPa, nearly the 75^*th*^ percentile of the sample (Fig 1b). Even when humidity was high – at the 99^*th*^ percentile (20hPa) – though significant, the marginal effect of heat on having been treated for anxiety or depression was only about 60% of the size of its effect on reporting high or very high distress.

**Fig 2:**
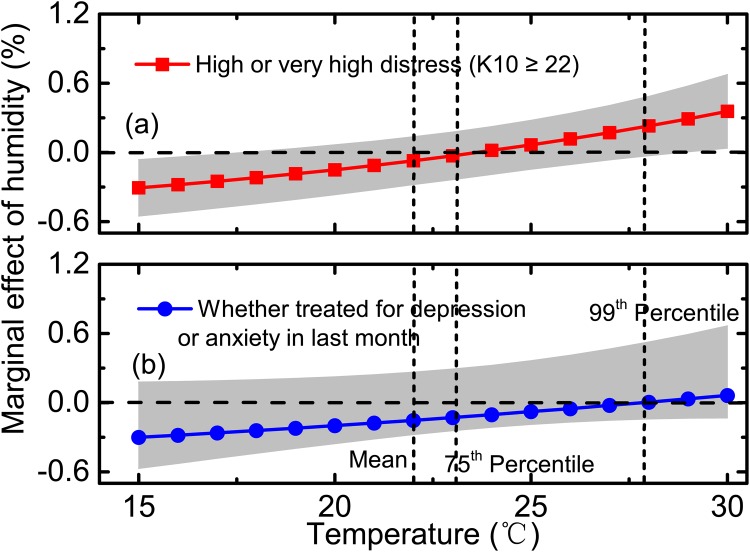
Marginal effects of humidity on high or very high distress and on receiving treatment for depression or anxiety by temperature and their 99% confidential intervals, Logit model (Model 4), 53,144 adults aged over 45 from NSW, Australia.

Fig 2 shows the increase in the marginal effect of humidity on high or very high distress (a) and on having been treated for depression or anxiety in the last month (b) as temperature increases. When the maximum average daily temperature was below 18°C, the marginal effect of humidity on distress was significant and negative. That is, the cooler the preceding four weeks, the weaker the association between humidity and distress. At the other end of temperature distribution, when average maximum monthly temperatures exceeded 29°C, the marginal effect of humidity on distress became significant and positive. At 30°C, a one-unit increase in humidity was associated with a 0.4% (*p* < 0.001, 99% CI: 0–0.7%) increase in the occurrence of high or very high distress.

Although the marginal effect of humidity on the likelihood of being treated for depression or anxiety followed the same pattern, rising monotonically with temperature, it was non-significant for the whole range of temperature.

The estimates in Table 3 reveal the non-linear nature of the associations between hot and/or humid weather and high or very high distress: as before, the higher the heat and/or humidity, the greater the increase in the likelihood of experiencing high or very high distress. Once the average daily temperature rose between 26.3 and 26.8°C (the 96-97^*th*^ percentile of the sample), the occurrence of high or very high distress increased by 2.0% (*p* < 0.01, 99% CI: 0.4-3.6%). When it reached between 26.8 and 27.8°C (the 98-99^*th*^ percentile of the sample), the corresponding estimated value rose to 2.6% (*p* < 0.001, 99% CI: 0.9-4.2%). A similar, though weaker, curvilinear relationship was evident for average monthly humidity. At the 90-95^*th*^ percentile (18.1-19.2 hPa), the occurrence of high or very high distress increased by 1%, though not significantly, rising to 2.0% (*p* < 0.01, 99% CI: 0.3-3.8%) when humidity reached the 96-97^*th*^ percentile (19.2-19.6 hPa) and rising to 2.3% (*p* < 0.001, 99% CI: 0.6-4.0%) at the 98-99^*th*^ percentile (19.6 and 20.4 hPa). Note that, at the highest percentiles for temperature and vapour pressure, the estimates are non-significant, likely due to the small cell size at this point.

**Table 3:** Non-linear associations between temperature (heat), vapour pressure (humidity) and mental health, Logit model, 53,144 adults aged over 45 from NSW, Australia.

	Percentile	High or very high distress (K10 ≥ 22)		Whether treated for depression or anxiety in last month	
		Coef. (99% CI)	M. E. (99% CI)	Coef. (99% CI)	M. E. (99% CI)
Model 1′^*a*^^*b*^, Heat (Ref. Temperature lower than 25.0°C, 90%)					
Temperature between 25.0 and 26.3°C	91-95	0.070 (-0.149 – 0.289)	0.4% (-0.8% – 1.5%)	0.098 (-0.126 – 0.323)	0.5% (-0.7% – 1.7%)
Temperature between 26.4 and 26.8°C	96-97	0.373 (0.067 – 0.680)*	2.0% (0.4% – 3.6%)*	0.164 (-0.183 – 0.510)	0.9% (-1.0% – 2.7%)
Temperature between 26.9 and 27.8°C	98-99	0.481 (0.167 – 0.794)**	2.6% (0.9% – 4.2%)**	0.112 (-0.260 – 0.484)	0.6% (-1.4% – 2.5%)
Temperature higher than 27.8°C	100	0.197 (-0.258 – 0.652)	1.0% (-1.4% – 3.5%)	-0.268 (-0.792 – 0.257)	-1.4% (-4.1% – 1.3%)
Model 2′^*a*^^*b*^, Humidity (Ref. Vapour pressure lower than 18.1 hPa, 90%)					
Vapour pressure between 18.1 and 19.2 hPa	91-95	0.184 (-0.032 – 0.399)	1.0% (-0.2% – 2.1%)	0.058 (-0.176 – 0.291)	0.3% (-0.9% – 1.5%)
Vapour pressure between 19.3 and 19.6 hPa	96-97	0.384 (0.060 – 0.709)*	2.0% (0.3% – 3.8%)*	0.036 (-0.334 – 0.405)	0.2% (-1.7% – 2.1%)
Vapour pressure between 19.6 and 20.4 hPa	98-99	0.430 (0.116 – 0.745)**	2.3% (0.6% – 4.0%)**	0.280 (-0.064 – 0.624)	1.5% (-0.3% – 3.2%)
Vapour pressure higher than 20.4 hPa	100	0.358 (-0.088 – 0.804)	1.9% (-0.5% – 4.3%)	-0.151 (-0.687 – 0.386)	-0.8% (-3.6% – 2.0%)

Notes:

^a^ All models control for illness history (physical and mental), age, age squared and the interactions between age and gender and the interaction between age squared and gender, urbanicity/remoteness, labour force participation status, highest level of educational attainment, relationship status and use of language other than English at home (as a proxy for cultural background).

^b^ Because of the large sample size, significance values were set at:

* *p* <.01,

** *p* <.001.

Again, the model predicting whether respondents had been treated for depression or anxiety shows that the estimated effects of both heat and humidity were non-significant.

## Discussion

In this study of 53,144 people aged 45 years and older living in New South Wales (NSW), Australia, we investigated how heat and humidity are associated with the likelihood of experiencing high or very high distress (which indicates the likely presence of mental disorders such as depression and anxiety) and of actually having been treated for depression or anxiety. We find that increases in both temperature and vapour pressure, when considered separately, are significantly associated with increases in the likelihood of reporting high or very high distress. When considering heat and humidity together, the estimate of vapour pressure is non-significant and its marginal effect at mean is nearly zero. So, heat, rather than humidity, may be the principal cause of high or very high distress, particularly when temperature is close to the sample mean (about 22°C). However, this finding is nuanced by the combined effect of heat and humidity in which rising humidity modifies the effect of heat on high or very high distress. When the weather is extremely humid, the harmful effect of increasing heat on distress is approximately doubled. Humidity itself has a slightly more complex relationship with distress. In hot months, when the average maximum temperature rises above about 29°C, high humidity is associated with (even) greater distress; but in cool months (when the average maximum temperature is below 18°C), high humidity is associated with less distress. We did not find these effects of heat or humidity on the likelihood of being treated for depression and anxiety.

The association between weather and some aspects of mental wellbeing has previously been investigated. For the most part, studies have focused on the harmful effect of heat or heat waves on outcomes such as mental health, vitality, role-emotional and social functioning [9], life satisfaction [24–27], feelings [26], happiness [26, 28, 29], and mood [30, 31]. To our knowledge, only two studies have considered how a variety of weather conditions is associated with mental health measured by life satisfaction [24, 26], one of which further examined the effect on happiness, positive/negative affect, tiredness and etc [26]. But, none has investigated the role humidity plays in the relationship between heat and mental health. This study provides confirmation of the harmful effect of heat on population mental health and adds information about how humidity affects the association between heat and mental health.

Although, as our estimates show, the marginal effects of heat and humidity are small, the implication of these effects is non-trivial. In NSW Australia, where the population aged over 45 was about 2.82 million at the time of this study [19], a one-degree increase in temperature would have been associated with almost 6,000 additional persons experiencing high or very high distress; and a one-unit increase in humidity with about 3,000 additional persons experiencing such distress. These numbers could increase substantially in the future, due to global warming, with harmful implications for human suffering and healthcare costs. First, as the earth warms, heat waves are expected to become more severe, more persistent and more frequent [32]. Second, between 2008 and 2013, the NSW population aged more than 45 years increased by nearly 10% [33]; this ageing trend is expected to continue and there is a similar trend globally [34]. Thus, in future, the relative and absolute numbers of middle-aged and older individuals whose mental health may be compromised by rising temperature and humidity could be much larger than we have estimated here.

One important implication of our findings relates to the fact that the likelihood of receiving treatment for depression or anxiety is not significantly correlated with heat and humidity. Thus, even though hot and humid conditions are harmful to mental health, the additional need of NSW residents exposed to such conditions was not being actively or systematically considered in health services planning. With about 8% of respondents state-wide having been treated for depression and anxiety in the last month but only 6.5% reporting high or very high distress (indicating likely presence of illness and, therefore, likely presence of treatment need), this statement may seem contradictory. However, it is more likely that it reflects a structural supply shortage in mental health care provision, a vital nuance that is lost when analysing population means. That is, treatment need in hot, humid months is greater than population need overall; but, while mental health service need increases in hot/humid months, respondents fail to seek help from health professionals (or health professionals fail to respond to the weather-related changes in need).

One possible weakness of this study is that SEEF data were collected over a relatively short period of time and the majority of participants completed their questionnaires during the spring season. It could therefore be argued that the weather conditions at that time (such as heat and humidity) are just a surrogate for geographic location (for example, inland or coastal areas), which could be the ‘real’ risk factor for mental health. Such an argument is not without precedent: economic structures in coastal areas differ from those in inland areas, which might cause the corresponding differences in mental health. To control for this possibility, our study included a variable indicating whether an area was coastal or not; this did not alter our findings (See S3 and S4 Tables). Another concern is that (few) total hours of daylight is a risk factor for mental health and our findings may thus have been driven by seasonality. However, we controlled in all analyses for the average solar exposure over the relevant timeframe (the last four weeks) and, again, while this slightly changed the magnitude of the effects, it did not affect the pattern of results (See S3 and S4 Tables). While we found no evidence of systematic bias with respect to these factors, this is worthy of more detailed study.

A further limitation of this study is that self-selection may have biased the estimates: people who tolerate hot weather poorly might choose to live in cooler places, or depart temporarily during very hot weather, perhaps leaving a possibly vulnerable group under-represented. However, any such bias would be downward, meaning that the estimates presented in this study would represent the lower bound of the effect of hot/humid weather on mental health. Relatedly, because data were collected in the springtime, few participants responded during extremely hot (or cold) and humid weather although 2007 has been the warmest year on record for New South Wales. The annual mean temperature for the state was 18.4°C, which is 1.1°C above the historical average of 17.3°C [35]. Considering the nonlinear nature of the association between heat and humidity and distress, the effect of extreme heat/humidity on mental health could be much greater than estimated here and should be tested on samples collected in summertime. A third limitation is that the participants in this study were aged at least 45 years and their reaction to heat/humidity may not reflect the impact of these weather conditions on younger people. Finally, we calculated meteorological conditions (temperatures and vapour pressures) at the centre of each statistical local area (SLA) as a proxy for the heat and humidity exposure of all participants in that area. This would have introduced some measurement error into the analysis, especially as, in Australia, some SLAs can be very large. However, more than 60% of the NSW population resides in metropolitan locations where SLAs are small and our estimation approach would introduce limited error. Furthermore, our estimates reflect the fact that, at present, the Australian health system does not moderate the provision of mental health care according to extreme meteorological conditions; thus the limited measurement error that may be present makes no material difference to our conclusions. Future studies will need to account for potential self-selection bias, greater variation in hot and cold weather conditions and younger age groups. Considering the adaptation capacity of human beings and locality of climate or weather, departures from the long-term mean of the weather conditions should also be the investigated. More sophisticated statistical models using longitudinal cohort data and weather condition modelling are also needed, as are more detailed models estimating the timing and magnitude of likely spikes in mental health service demand.

Despite these limitations, the findings of this study are underpinned by excellent epidemiological and weather data. We had access to a very large and geographically dense sample (more than 53,000 respondents living in a single Australian state), which represents 1.9% of the NSW population aged 45 years and older. As NSW contains a range of climate zones, we had access to sufficient (if not excellent) variance in respondents’ exposure to heat and humidity. Unlike most studies, the daily weather data we used were produced at a very fine-grained resolution (about 5km × 5km). Because we knew the date on which participants’ mental health was reported and where they lived, we could match their exposure to heat and humidity closely. Furthermore, we minimised the bias associated with the inherent relationship between heat and humidity [12] and the possible effects of each on mental health by examining heat (and its interaction with humidity) separately and together in our equations. The wealth of socio-economic and demographic information available in the dataset enabled us to control for a very a wide array of potentially confounding factors which have previously been demonstrated to be closely related to health status and which could have biased our estimates significantly.

We acknowledge that we may have underestimated the effect of hot/humid weather on mental health, but this does not undermine the conclusion that humidity aggravates the negative effect of heat on mental health. To the contrary, the effects may be stronger than the estimates reported here. In the context of climate change, health authorities are not only likely to face growth in total mental health care demand but will also have to improve their capacity to supply care nimbly to different localities with varying levels of need. To promote overall mental health and resilience, increased public discussion is urgently required to help citizens work together with service providers to protect and promote mental health as the planet warms [36].

Because the impacts of weather on mental health are complicated to explain, public health officials also need easy access to simple but reliable indices or early warning systems – an urgent priority for academic-policy co-research. There is also a significant leadership role for mental health professionals, such as psychiatrists, as well as general practitioners [37], who should join the effort to alert politicians, authorities and the public to this challenge and to the pressing need to shape effective responses; a role that must be supported by professional colleges around the world [38, 39].

## Supporting Information

S1 Table The distribution of participants over months of the year. (DOCX)

S2 Table Robustness check 1, the associations between temperature (heat), vapour pressure (humidity) and mental health, Logit model for 53,144 adults aged over 45 from NSW, Australia. (DOCX)

S3 Table Robustness check 2 and 3, the associations between temperature (heat), vapour pressure (humidity) and mental health, Logit model for 53,144 adults aged over 45 from NSW, Australia. (DOCX)

S4 Table Robustness check 2 and 3, the non-linear associations between temperature (heat), vapour pressure (humidity) and mental health, Logit model, 53,144 adults aged over 45 from NSW, Australia. (DOCX)

S1 Fig The mean K10 score by month of the year when participants completed questionnaires, 53,144 adults aged over 45 from NSW, Australia. (EPS)
