# Supplementary material for: The Importance of Humidity in the Relationship between Heat and Population Mental Health: Evidence from Australia
Source: PLoS One. 2016 Oct 11;11(10):e0164190. doi: 10.1371/journal.pone.0164190 (PMC5058549; doi:10.1371/journal.pone.0164190)
Supplement: S1 Table — (DOCX) [file pone.0164190.s001.docx]

| Month of the year | Observations | |
| --- | --- | --- |
|  | n | % |
| Jan | 18 | 0.03 |
| Feb | 5 | 0.01 |
| Mar | 1,378 | 2.59 |
| Apr | 735 | 1.38 |
| May | 135 | 0.25 |
| Jun | 374 | 0.70 |
| Jul | 1,398 | 2.63 |
| Aug | 118 | 0.22 |
| Sep | 60 | 0.11 |
| Oct | 33,787 | 63.58 |
| Nov | 9,913 | 18.65 |
| Dec | 5,223 | 9.83 |

Table S1 The distribution of participants over months of the year.
