# Supplementary material for: The Importance of Humidity in the Relationship between Heat and Population Mental Health: Evidence from Australia
Source: PLoS One. 2016 Oct 11;11(10):e0164190. doi: 10.1371/journal.pone.0164190 (PMC5058549; doi:10.1371/journal.pone.0164190)
Supplement: S2 Table — (DOCX) [file pone.0164190.s002.docx]

|  | High or very high distress (K10 ≥ 22) | | Whether treated for depression or anxiety in last month | |
| --- | --- | --- | --- | --- |
|  | Coef. (99% CI) | M.E. (99% CI) | Coef. (99% CI) | M.E. (99% CI) |
| Model 1 | | | | |
| Temperature | 0.027 (0.007 - 0.046)** | 0.1% (0.000 - 0.002)** | 0.000 (-0.020 - 0.020) | 0.000 (-0.001 - 0.001) |
| Model 2 | | | | |
| Vapour pressure | 0.027 (0.006 - 0.049)** | 0.1% (0.000 - 0.002)** | -0.086 (-0.232 - 0.061) | 0.000 (-0.001 - 0.001) |
| Model 3 | | | | |
| Temperature | 0.024 (-0.026 - 0.074) | 0.1% (-0.001 - 0.004) | 0.009 (-0.042 - 0.060) | 0.000 (-0.002 - 0.003) |
| Vapour pressure | 0.003 (-0.051 - 0.057) | 0.0% (0.003 - 0.003) | -0.011 (-0.067 - 0.045) | -0.001 (-0.003 - 0.002) |
| Model 4 | | | | |
| Temperature | -0.066(-0.146 - 0.014) | 0.3%(0.000 - 0.006)* | -0.048 (-0.130 - 0.035) | 0.002 (-0.001 - 0.005) |
| Vapour pressure | -0.185(-0.332 - -0.038)* | -0.2%(-0.005 - 0.001) | -0.133 (-0.285 - 0.020) | -0.002 (-0.005 - 0.001) |
| Temperature × Vapour pressure | 0.009(0.002 - 0.015)* |  | 0.006 (-0.001 - 0.012) |  |

Table S2 Robustness check 1, the associations between temperature (heat), vapour pressure (humidity) and mental health, Logit model for 53,144 adults aged over 45 from NSW, Australia.

Notes:

1. All models control for illness history (physical and mental), age, age squared and the interactions between age and gender and the interaction between age squared and gender, urbanicity/remoteness, labour force participation status, highest level of educational attainment, relationship status and use of language other than English at home (as a proxy for cultural background).
2. Temperature and vapour pressure are the average of the daily average temperature and the average vapour pressure in the last 28 days respectively;
3. Because of the large sample size, significance values were set at: * *p* <.01, ** *p*<.001.
