# Supplementary material for: The Importance of Humidity in the Relationship between Heat and Population Mental Health: Evidence from Australia
Source: PLoS One. 2016 Oct 11;11(10):e0164190. doi: 10.1371/journal.pone.0164190 (PMC5058549; doi:10.1371/journal.pone.0164190)
Supplement: S3 Table — (DOCX) [file pone.0164190.s003.docx]

|  | High or very high distress (K10 ≥ 22) | | Whether treated for depression or anxiety in last month | |
| --- | --- | --- | --- | --- |
|  | Specification I | Specification II | Specification I | Specification II |
|  | Coef. (99% CI) | Coef. (99% CI) | Coef. (99% CI) | Coef. (99% CI) |
| Model 1 | | | | |
| Temperature | 0.031 (0.016 - 0.047)** | 0.030 (0.011 - 0.048)* | 0.005 (-0.011 - 0.021) | -0.005 (-0.024 - 0.014) |
| Model 2 | | | | |
| Vapour pressure | 0.033 (0.016 - 0.050)** | 0.024 (0.007 - 0.041)* | -0.0001 (-0.018 - 0.017) | -0.008 (-0.026 - 0.009) |
| Model 3 | | | | |
| Temperature | 0.022 (-0.007 - 0.050) | 0.026 (-0.006 - 0.057) | 0.018 (-0.012 - 0.047) | 0.007 (-0.026 - 0.039) |
| Vapour pressure | 0.013 (-0.019 - 0.044) | 0.005 (-0.025 - 0.034) | -0.017 (-0.050 - 0.016) | -0.014 (-0.044 - 0.017) |
| Model 4 | | | | |
| Temperature | -0.081(-0.148 - 0.015) | -0.088(-0.158 - -0.019) | -0.045 (-0.113 - 0.023) | -0.064 (-0.136 - 0.007) |
| Vapour pressure | -0.189(-0.312 - -0.067)* | -0.202(-0.319 - -0.085)** | -0.141 (-0.269 - 0.014) | -0.144 (-0.267 - 0.022) |
| Temperature × Vapour pressure | 0.008(0.003 - 0.013)** | 0.009(0.004 - 0.013)** | 0.005 (0.000 - 0.010) | 0.005 (0.001 - 0.010) |

Table S3 Robustness check 2 and 3, the associations between temperature (heat), vapour pressure (humidity) and mental health, Logit model for 53,144 adults aged over 45 from NSW, Australia.

Notes:

1. All models control for illness history (physical and mental), age, age squared and the interactions between age and gender and the interaction between age squared and gender, urbanicity/remoteness, labour force participation status, highest level of educational attainment, relationship status and use of language other than English at home (as a proxy for cultural background).
2. Specification I controls for whether the SLA is coastal; Specification II controls for the average sun exposure in last four weeks;
3. Because of the large sample size, significance values were set at: * *p* <.01, ** *p*<.001.
