# Supplementary material for: The Importance of Humidity in the Relationship between Heat and Population Mental Health: Evidence from Australia
Source: PLoS One. 2016 Oct 11;11(10):e0164190. doi: 10.1371/journal.pone.0164190 (PMC5058549; doi:10.1371/journal.pone.0164190)
Supplement: S4 Table — (DOCX) [file pone.0164190.s004.docx]

|  | High or very high distress (K10 ≥ 22) | | Whether treated for depression or anxiety in last month | |
| --- | --- | --- | --- | --- |
|  | Specification I | Specification II | Specification I | Specification II |
|  | Coef. (99% CI) | Coef. (99% CI) | Coef. (99% CI) | Coef. (99% CI) |
| Model 1’, Heat (Ref. Temperature lower than 25.0 ℃, 90%) | | | | |
| Temperature between 25.0 and 26.3 ℃ | 0.071 (-0.095 - 0.238) | 0.046 (-0.125 - 0.217) | 0.101 (-0.070 - 0.272) | 0.068 (-0.107 - 0.244) |
| Temperature between 26.4 and 26.8 ℃ | 0.373 (0.139 - 0.606)* | 0.354 (0.118 - 0.589)* | 0.162 (-0.101 - 0.426) | 0.139 (-0.126 - 0.405) |
| Temperature between 26.9 and 27.8 ℃ | 0.480 (0.241 - 0.718)** | 0.462 (0.221 - 0.702)** | 0.111 (-0.173 - 0.394) | 0.087 (-0.198 - 0.373) |
| Temperature higher than 27.8 ℃ | 0.194 (-0.152 - 0.541) | 0.172 (-0.176 - 0.521) | -0.271 (-0.670 - 0.128) | -0.298 (-0.699 - 0.103) |
| Model 2’, Humidity (Ref. Vapour pressure lower than 18.1 hPa, 90%) | | | | |
| Vapour pressure between 18.1 and 19.2 hPa | 0.190 (-0.025 - 0.354) | 0.169 (0.002 - 0.336) | 0.062 (-0.115 - 0.240) | 0.034 (-0.146 - 0.215) |
| Vapour pressure between 19.3 and 19.6 hPa | 0.394 (0.146 - 0.641)* | 0.366 (0.116 - 0.616)* | 0.042 (-0.239 - 0.324) | 0.006 (-0.278 - 0.290) |
| Vapour pressure between 19.6 and 20.4 hPa | 0.444 (0.203 - 0.684)** | 0.412 (0.170 - 0.655)** | 0.289 (-0.027 - 0.552) | 0.250 (-0.015 - 0.515) |
| Vapour pressure higher than 20.4 hPa | 0.380 (-0.038 - 0.721) | 0.351 (0.011 - 0.691) | -0.134 (-0.544 - 0.275) | -0.162 (-0.570 - 0.246) |

Table S4 Robustness check 2 and 3, the associations between temperature (heat), vapour pressure (humidity) and mental health, Logit model for 53,144 adults aged over 45 from NSW, Australia.

Notes:

1. All models control for illness history (physical and mental), age, age squared and the interactions between age and gender and the interaction between age squared and gender, urbanicity/remoteness, labour force participation status, highest level of educational attainment, relationship status and use of language other than English at home (as a proxy for cultural background).
2. Specification I controls for whether the SLA is coastal; Specification II controls for the average sun exposure in last four weeks;
3. Because of the large sample size, significance values were set at: * *p* <.01, ** *p*<.001.
